# Barriers to Condom Use among High Risk Men Who Have Sex with Men in Uganda: A Qualitative Study

**DOI:** 10.1371/journal.pone.0132297

**Published:** 2015-07-14

**Authors:** Geofrey Musinguzi, Hilde Bastiaens, Joseph K. B. Matovu, Fred Nuwaha, Geoffrey Mujisha, Juliet Kiguli, Jim Arinaitwe, Jean-Pierre Van Geertruyden, Rhoda K. Wanyenze

**Affiliations:** 1 Disease Control and Environmental Health, Makerere University School of Public Health, Kampala, Uganda; 2 International Health, University of Antwerp, Antwerp, Belgium; 3 Primary and Interdisciplinary Care University of Antwerp, Antwerp, Belgium; 4 Community Health and Behavior Sciences Makerere University School of Public Health, Kampala, Uganda; 5 MARPS Network, Kampala, Uganda; 6 Ministry of Health, Kampala, Uganda; David Geffen School of Medicine at UCLA, UNITED STATES

## Abstract

**Background:**

Unprotected sexual intercourse is a major risk factor for HIV transmission. Men who have sex with men (MSM) face challenges in accessing HIV prevention services, including condoms. However, there is limited in-depth assessment and documentation of the barriers to condom use among MSM in sub-Saharan Africa. In this paper, we examine the barriers to condom use among MSM in Uganda.

**Methods:**

The data for this study were extracted from a larger qualitative study conducted among 85 self-identified adult (>18 years) MSM in 11 districts in Uganda between July and December 2013. Data on sexual behaviours and access and barriers to condom use were collected using semi-structured interviews. All interviews were audio-recorded and transcribed verbatim. This paper presents an analysis of data for 33 MSM who did not use condoms at last sex, with a focus on barriers to condom use. Analysis was conducted using the content analysis approach.

**Results:**

Six major barriers to condom use were identified: Difficulties with using condoms, access challenges, lack of knowledge and misinformation about condom use, partner and relationship related issues, financial incentives and socio-economic vulnerability, and alcohol consumption.

**Conclusion:**

The findings suggest that several reasons account for lack of condom use among high-risk MSM. The findings are valuable to inform interventions needed to increase condom use among MSM.

## Introduction

Studies from low, middle and high-income countries indicate a high risk of HIV transmission among men who have sex with men (MSM) [1–3]. Studies in the United States show that some MSM engage in high risk sexual behaviors such as unprotected receptive and insertive anal sex, and multiple sexual partnerships in the absence of consistent condom use [2–4]. Some MSM may also engage in drug and alcohol abuse prior to sex, which impairs judgment and increases the likelihood of unprotected anal intercourse, sometimes with people of unknown HIV sero-status [2–4]. It is well known that multiple sexual partners and high-risk sexual behaviours such as unprotected penile-anal sexual intercourse increase the risk of HIV acquisition [5–7]. In a study in Mombasa Kenya, MSM had the belief that having anal sex was less risky for HIV acquisition than having vaginal sex [8]. Yet, a recent study showed that the risk of HIV infection through anal sex was about 18 times higher than through vaginal sex [9].

The prevalence of HIV among MSM is higher than that in the general population, and ranges between 11% in the Caribbean, 25% in Africa, 28% in Southeast Asia and 51% in parts of Latin America [10]. While Uganda’s HIV epidemic is generalized with all communities affected [11], recent studies show that HIV prevalence among MSM is about twice as in the general population (13.5% compared to 7.3%) [12].

Although not widespread, interventions to reduce sexual risk-taking behaviors among MSM in Uganda have focused on harm reduction strategies such as condom use and reduction of multiple sexual partners. Condom use presents the most credible HIV prevention strategy available to high risk MSM, but evidence elsewhere shows limited usage among the MSM populations [11].

Several reasons have been documented to explain the limited condom use among MSM including preference for condom-less sex, low HIV risk perception, context, relationships and interpersonal communication [13–15]. However, most of these studies were conducted in high and middle-income countries. In sub-Saharan Africa including Uganda, same-sex behaviours have been largely neglected by HIV research [16] mainly due to the restrictive legal environment and severe stigma [17–18] leaving significant knowledge gaps in terms of in-depth understanding of the barriers to condom use among MSM.

MSMs in Uganda are highly closeted (i.e. hidden) and thus face an increased risk of contracting HIV relative to the exclusively heterosexual persons [5, 19]. Understanding the barriers to condom use among high risk MSM is critical to the development of targeted HIV preventive strategies for this community. The purpose of this study was to explore the phenomenon of non-condom use and specifically focus on barriers to condom use among high risk MSM in Uganda whom we defined as males who had penetrative penile-anal sex with other males, regardless of sexual orientation or gender identity [20].

## Methods

### Setting

The data for this study were extracted from a larger qualitative study conducted among 85 self-identified adult MSM (≥18 years) in 11 districts in Uganda. With the exception of one district, 8 MSM were recruited in each district, for the larger study. Main findings from this study are reported elsewhere [21]. The 11 districts were; Kampala, Mukono, Rakai in Central Uganda; Busia, Iganga, Mbale, Soroti in the Eastern region; Gulu in Northern Uganda; and Mbarara, Hoima and Bushenyi in Western Uganda. The selection of the districts took into consideration geographical representation, HIV prevalence, and existence of known hotspots for high risk groups such as sex workers and MSM. Most of the districts that were selected lie along the transport corridors known for high concentration of mobile and high-risk populations [22].

### Study design and setting

This was a cross-sectional descriptive study that used qualitative methods of data collection. The data were collected using semi-structured interviews with open-ended questions and probes to gather information. Choosing diverse settings as described above, we aimed ad recruiting a varied sample of participants. One to two seeds per district were chosen purposefully. Further recruitment happened via the snowballing technique. We opted for this approach since the population we wanted to include is not easily accessible [23]. Of the 85 self-identified adult MSM, 33 reported that they did not use condoms at the last sexual intercourse. The latter, constituted the population for this study.

### Data collection

Data were collected by graduate research assistants who underwent a rigorous and extensive 7-day training to standardise data collection procedures. The training entailed a review of the study objectives, the legal context and its implications for confidentiality of research participants, interpersonal issues; ethics, confidentiality, and informed consent; seminars on interviewing techniques for key populations, and detailed instructions on administering the semi-structured interview guides. The use of semi-structured interview guides enabled data gathering on sexual behaviours, and barriers to accessing HIV services, including barriers to condom use for HIV/STI prevention. Interviews lasted between 60–90 minutes. All interviews were audio-recorded with consent from the participants. Of each participant socio-demographic characteristics were obtained via a short structured section of the questionnaire (age, education, work, religion, and relationship status).

### Data management

All qualitative data were transcribed verbatim and data in other languages were translated into English. Each transcript was reviewed by at least two people and transcripts in other languages were reviewed by research assistants fluent in both English and the local language. Data were organized with the help of Atlas.ti version 7, qualitative data management software.

### Data analysis

In order to identify the barriers to condom use among MSM in Uganda, datasets for 33 respondents who reported non-use of condoms at last sexual intercourse were sorted and analysed using content analysis with the help of Atlas.ti. Content analysis provides a theoretical framework to understand actual content. It is used to develop objective inferences about a subject of interest or determine certain words, phrases, characteristics or sets of texts in an objective manner [24]. Using open coding, all text related to barriers to condom use was coded into sub-categories by three investigators (GM, HB and RW). The sub-categories were further organized into families to form categories. For quality control, and to ensure that the interpretation was close to the content, the researchers kept a back-and-forth constant comparison of the categories and sub-categories with the transcripts [25]. Regular discussions among the coders further supported reflexivity on the analysis process and the emerging results.

### Ethics statement

Ethical approval was granted by Makerere University School of Public Health Higher Degrees-Research and Ethics Committee and by the Uganda National Council for Science and Technology. Each participant provided written informed consent. To ensure confidentiality, we used initials or pseudonyms as opposed to signatures or thumbprints. Confidentiality for all study participants was observed by conducting interviews in places with adequate privacy. These places were selected by the respondents. Study participants were issued with a written informed consent detailing the study, the risks and benefits, and emphasis on the protection of confidentiality.

## Results

### Characteristics of the study population

The participants’ age ranged from 18 to 40 years. Overall, 57% (48) had attained secondary level education, 40% (34) were Catholic, and 65% (55) had some form of employment. The majority (77%; 65) were in sexual relationships with male partners, while 17% (14) were in sexual relationships with female partners. About 9% were married to a female partner. Thirty eight per cent (33) reported that they did not use condoms at last sexual intercourse, and are the focus of this paper.

### Barriers to condom use among MSM

Six major categories related to non-use of condoms were identified: a) practical difficulties with using condoms, b) access challenges, c) lack of knowledge and misinformation about condom use, d) partner and relationship-related factors, e) financial incentives and socio-economic vulnerability and f) alcohol consumption. The following sub-section presents the study findings arranged by category.

### a) Practical difficulties with using condoms

MSM raised two main difficulties to explain why they did not use condoms at last sexual contact: (i) concerns about the quality of condoms and lack of lubricants; and (ii) discomfort & pain when using condoms, as shown in the following subsections:

#### Concerns about the quality of condoms and lack of lubricants

Participants perceived available male condoms as being of poor quality, expired or/and unsuitable for anal sex. For example, one of the participants from Mbarara reported; “*we had ‘engabo’ [condom type] they were telling us that they are not safe*, *you know all those issues*, *we heard complaints about [these] condoms*, *so I believe not using a condom*, *may be better*”. Another from Kampala narrated “*Sometimes they break … Some are not of good quality*, *that is; anal sex is a bit rough so we need to [have] a good quality condom … because these things are a bit rough”*. Most of the respondents who raised quality concerns had attempted to use condoms but experienced condom bursts during sexual encounters as elaborated by the following participant from Kampala. “*However much I try like doing protected sex [using condoms]…they all burst … anal sex is a bit rough so we need to [have] good quality condoms*”. In another related quote this respondent stated “*Any way we also just use condoms to protect lives but it is so painful*. (IDI 5, Bushenyi)”. In such circumstances (when condoms have burst) the usual tendency is to continue with unprotected sex, thereby increasing the possibility of HIV acquisition/transmission. One respondent said that three condoms ruptured in a single sexual encounter and described the experience as ‘un-enjoyable’. Respondents attributed the bursting of condoms to rough anal sex and/or insufficient or poor quality lubricants. Many respondents reported challenges with access to lubricants and improvised with alternatives such as jellies, oils and a few isolated cases reported using soap and saliva.

#### Discomfort and pain when using condoms

The physical pain caused by condoms during anal sex was noted as a barrier to condom use by some study participants. Some participants reported experiencing irritation and others suffered physical pain and bruises. “*The use of a condom is not bad but the problem is when used for more than a minute*, *it tends to get dry and it starts hurting and even it can create bruises*. *It can only be good when one uses it for a few minutes*, *then get a new one*” (IDI 3, Soroti). One participant said that he tried changing the condom more frequently in an attempt to reduce the pain and discomfort. However, he noted that this would make the process “tedious and not enjoyable”.

### b) Access challenges

MSM cited difficulties in accessing condoms as another barrier to condom use. They said that sometimes condoms are out of stock and in some instances they are not sold in some of the areas where they reside. This barrier was more commonly cited by participants in the rural areas. One of the subjects from a rural district lamented that if he had received condoms and used them the last time he had sex, he would never have suffered the ulcer that he developed following unprotected anal sex. “*Because these things you see here [condoms] I even never had them*, *I got this one [ulcer] from …; because if I had them I wouldn’t experience that damage*” (IDI 6, Iganga)

Although not commonly mentioned, affordability was also highlighted by some respondents as a barrier to condom use. Some respondents reported that they never used condoms either because they didn’t have money to buy them or they lacked both money and the place to acquire them from.

### c) Lack of knowledge and misinformation about condom use

Another barrier to condom use was the apparent lack of knowledge about condoms or their use that was exhibited by MSM coupled with the misinformation that they had regarding condom use. Lack of knowledge was manifested in various forms including: lack of knowledge on how to use condoms; condom-related fears and homophobia, as well as the belief that sex with a fellow man is safe, as shown below.

#### Lack of knowledge about condoms or how to use condoms

Despite widespread knowledge about condoms in the general population, some participants reported that they didn’t know anything about condoms while others reported that they didn’t know how to use them. One respondent said he didn’t know anything about condoms. Another respondent said he wasn’t quite sure of how condoms work while another said he had never had experience of using condoms. The gaps in the know-how of using condoms are further demonstrated by a respondent who said that he was in possession of condoms but he feared to use them since he did not know how to use them. “*I don’t quite understand how they [condoms] work*” (IDI 8, Mbarara). Another participant also related, “*I even have some condoms which I bought recently …I actually want to learn those things but still I fear*” (IDI 2, Bushenyi).

#### Condom-related fears and homophobia

This sub-category reveals multiple facets that guide the decision-making process and why some MSM chose not to use condoms. Participants tended to have a series of unanswered questions regarding condom use: ‘What if the condom got stuck into them; where would they go for help?’ ‘How would they start explaining?’ One participant said, “*…and when you are taken for treatment*, *what will you say*? *They [health workers] will ask you*, *“How has it [condom] entered*? *What have you been doing*?” (IDI 1, Bushenyi). Visiting a healthcare facility for such a problem would reveal their sexual practices. Thus, the fear of negative consequences resulting from use of condoms offset the concerns about the risk of not using them. “*…I just don’t like using condoms because when I heard of cases where a condom could stick in the anus. … such things make me not like condoms and imagine going to the doctor who is not used to such things*” (IDI 7, Kampala).

#### Belief that sex with a fellow man is safe

Some participants who reported non-use of condoms at last sex particularly from Iganga, Rakai, and Hoima districts reasoned that they did not need to use condoms when having sex with fellow men since it is only sex with women that can result into HIV transmission or acquisition. When asked why he didn’t use a condom, one of the respondents said “*because I was not having sex with a woman*” (IDI 7, Iganga). Another respondent said: “*But when having sex with a fellow man … it is only one person releasing the sperms you have no chances of getting infected with HIV*” (IDI 2, Iganga). This perception is further supported by the following articulations from another respondent:

“There are those also who think that someone having sexual intercourse with a fellow man cannot get HIV with the reasoning that the man does not have the fluid like the ladies and yet they have always known that it is transmitted through [vaginal] fluids so how do they get it?” (IDI 1, Mbale).

Some participants reported that they were told, by other men within their networks, not to use condoms. One of the respondents said that they were told in their group that condoms do not prevent HIV, and are only meant to prevent pregnancies. He further narrated that in order not to contract HIV; they were told to have sex with only fellow men and not with women. When probed further, this participant felt very confident that it was not necessary to use a condom for protection since he did not have sex with women.

“we hear over the radios people saying condoms cause diseases … they [other people within their networks] warn us in advance that we shouldn’t lie to ourselves that we won’t catch HIV when we use condoms when having sex with women … instead we will acquire it”. “Condoms can’t prevent HIV but they can prevent pregnancy in case you have sex with a woman but they can’t prevent HIV” (IDI 2, Iganga).

### d) Partner and relationship related factors

This category is based on four sub-categories: trusting the partner coupled with awareness of each other’s HIV status; weak safer sexual negotiation skills; non-cooperative and sometimes violent partners; and unplanned sexual encounters and attractiveness of the partner. The following sub-sections present an overview of the findings under each sub-category.

#### Trusting the partner coupled with awareness of each other’s HIV status

Participants reported that the trust that partners have for each other coupled with awareness of each other’s HIV status can be a great barrier in using condoms during anal sex. One participant said, “*Partners simply do not want condoms because they reason that a couple that uses a condom has no faith in each other*” (IDI 4, Hoima). Trust was linked to the length of the relationship with non-use of condoms commonly cited in long-term relationships. Some participants said that they used condoms at the beginning of the relationship but over time they didn’t see any reasons to continue using them, as this participant from Mbarara district intimated;

“Sometimes when you get together with someone and you have been together for a while you find no need of using a condom” (IDI 8, Mbarara)

The issue of trust between partners was more emphasized in MSM who have tested together and know each other’s HIV status. Once these partners became aware of each other’s HIV status, they stopped using condoms altogether. One participant from Mbarara had this to say: “*No [condom] because I have been with him for two and a half years; at first we were using condoms but then we had to go for an HIV test so after that test we tested negative so we trust each other*. *Like after three months*, *we go for a test*” (IDI 7, Mbarara)

#### Weak safer sexual negation skills

Trust or the lack of it was reported to influence the partners’ ability to negotiate for condom use. In situations where partners trusted each other, condom use was less likely. For example, one of the participants said that when his partner told him that he was safe, he decided to trust him; and because of this trust, he did not ask his partner to use a condom. Another participant said that when he tried to negotiate for safer sex, in response, his partner felt his comment meant he did not trust him anymore; to keep the relationship going, he gave in to unprotected sex. Some participants reported that they normally negotiate for safe sex but their partners reverse their decisions at the time of sexual intercourse. This challenge was most common among MSM who were engaged in commercial sex work.

“I remember there is a time I tried to speak about using it [condom] then he was like objecting to it; he asked me that “you no longer trust me nowadays?” and because I love him I have to accept to go without any protection” (IDI 4, Mbale).

#### Unplanned sexual intercourse

Lack of prior preparation for sex was reported to be one of the major hindrances to condom use especially when subjects were caught up in the ‘heat of the moment’. Some participants stated that condoms were not available at the time; others stated that they were not prepared and another one referred to it as “*the only mistake he has ever made*”. In addition, some respondents indicated that they had nowhere to buy condoms especially at night: ‘*Sometimes it happens let’s say in the night when you do not have condoms in the house so you end up doing it*’ (IDI 7, Kampala)

“We had nowhere to buy condoms from. There are times when this person comes for you when both of you aren’t prepared for it [sex]” (IDI 5, Bushenyi).

#### Attractiveness of the partner

Although this wasn’t very common, it was cited as a factor for non-use of condoms. The respondents said that sometimes they get attracted to the physical appearance of their partners; they reported that the male partner may appear tempting to have sex with–that is, ‘masculine’ and ‘well-built’, and therefore not likely to be infected with HIV.

“You may admire him because he is handsome and you decide to do it [sex] live thinking that he may be negative” (IDI 1, Hoima)

### e) Financial incentives and socio-economic vulnerability

Financial incentives were cited as a strong factor especially among men who were engaged in commercial sex. The male sex workers were more likely to give in when the client negotiated a higher pay for unprotected sex. Financial challenges appeared to be an underlying factor in this group and some survived on sex work as their only source of income. One participant said that when the financial offer on table is evaluated against the needs (rent, clothes, and food) coupled with the low turnover of clients and the risk, the financial offer overrides the risk. Thus, they give in to unprotected sex.

“You may find that he tells you I am paying you a lower price with a condom on and higher price with a condom off… and you may find that you are having that client today but it will take you something like a week so you think about that and say you need the money so you will be forced to go for the bigger amount. And remember I have very many needs I have to pay my rent dress up and eat” (IDI 5, Busia).

“…but if the customer does not want to use a condom I charge him higher–between 250,000/ = [USD 100] and 300,000/ = [USD 120] and with a condom on it is between 150,000/ = [USD 60] and 180,000/ = [USD 72]” (IDI 2, Gulu).

### f) Alcohol and other factors

Alcohol use was cited as a key barrier to condom use coupled with other barriers such as the need to maximize sexual pleasure, the need to “feel someone better” and being in a hurry, as summarized below.

#### Alcohol use

Alcohol use was cited as one of the key causes of non-use of condoms. Participants said that alcohol use influences their sexual behaviour decisions. Participants noted that being high on alcohol or drugs increases sexual urge and yet, when high, it is difficult to maintain self-control and think about condoms when having sex.

“There are times when you take a lot of alcohol, and when you take a lot of alcohol you will become very sexually active and may not even think of using a condom” (IDI 2, Busia).

“Some MSMs use drugs and alcohol which affect their normal reasoning capacity” (IDI 4, Hoima).

#### Sexual pleasure, exploration and being in a hurry

For some participants, non-use of condoms was attributed to the need to maximize sexual pleasure, and for others it was curiosity, interest, and uncontrollable desire. One of the participants compared a protected sexual encounter to eating a sweet in its cover. Another one said he was more comfortable and “felt the person well” without a condom. Others said that they were sometimes in a hurry and could not put on a condom.

“Another thing is that when I use a condom, I’m not comfortable when having sex. And I don’t feel the person well. And actually many people don’t want to use condoms because they don’t feel well with it while having sex. And with a condom on, I don’t enjoy sex” (IDI 3, Soroti).

## Discussion

This study of barriers to condom use among high risk Ugandan MSM found Six main barriers to condom use: (i) Difficulties using condoms; (ii) access challenges (iii) lack of knowledge and misinformation about condom use; (iv) partner and relationship factors; (v) financial incentives and socio-economic vulnerability and (vi) alcohol use and other factors. These findings provide additional evidence to that already reported in high and middle-income countries and is critical for designing interventions to increase condom use among high risk MSM in Uganda and other countries within sub-Saharan Africa [2–4, 15, 26–28].

Various factors including perceived condom quality, prior condom use experience, access to lubricants and access to condoms appear to interact closely to influence non-use of condoms. For example, the concerns about the quality of condoms is an important issue but, it appears to be related to other problems such as condoms breaking during sex, anal sex being rough, insufficient or inappropriate lubricants, bruises, pain, and the know-how of using condoms. In our study, access to lubricants was reported to be extremely difficult. Lubricants make sex safer by reducing the likelihood of condom breakage, and tissue damage and tearing caused to the genitals [20, 27]. The population we studied seems to understand the role of lubricants especially in prevention of bruises, discomfort and injuries during anal sex. However, they may not realize that the improvised lubricants are not designed to be used for anal sex with condoms; the possible reason as to why they develop bruises, injuries and discomfort. Hence, as a way out of pain and discomfort, they get rid of condoms. Besides, improvised lubricants are not recommended for anal sex. In another study that reported use of saliva, the authors highly criticized saliva usage because of the risk of other diseases that can be transmitted through saliva such as hepatitis B virus, and herpes virus [20]. Inadequacy and shortage of condoms also contributed to non-use of condoms. These challenges were compounded by high condom costs, heat of the moment and lack of availability of condoms within proximal distance.

Despite widespread campaigns about condoms in the general population, it was surprising to find that some MSM were unaware of and didn’t know how to use condoms. Furthermore, the belief among some MSM that having sex with fellow men has low risk of HIV transmission or acquisition was quite concerning. This perception that anal sex has low risk of HIV transmission compared to other forms of sex has been reported elsewhere [8]. Participants argued that, in vaginal sex, two people release sexual fluids whereas, in anal sex, one person releases sexual fluids, the reason for low risk of HIV transmission. Given that anal sex is also reported in heterosexual relations, and increasingly being reported among female sex workers [28–29], there is urgent need to reconsider condom promotion messaging. Messages such as “Having unprotected anal or vaginal sex exposes you to risk of HIV infection and the risk is higher with anal sex [9]” would counter the myths and false messages. Such messages could be delivered by trained peers, given the stigma and discrimination associated being MSM.

Non-use of condoms was also reportedly common among MSM in commercial sex industry and in long-term relationships. Reasons for non-use were attributed to financial incentives, coercion, and violence in commercial sex industry and trust and intimacy for those in long term relationships. These findings are not isolated; they have been reported by other studies among MSM and other sub-populations including female sex workers and persons living with HIV and AIDS [14, 30].

In their study, Ostergren asserts that some individuals enter a sexual experience with negative preconceived ideas about condoms based on past experience and personal preference [13]. In the same study, Ostergren reported that the ‘insertive’ partner never wanted to use condoms because they were aware that being insertive is associated with reduced risk of HIV transmission. Although this is true [31], available evidence suggests that the difference in risk between the two groups fades if the insertive partner is not circumcised [6].

Alcohol also appears to be a key determinant of non-use of condoms among MSM. The relationship between alcohol and risky sexual behaviour is well documented and cannot be over emphasized [13, 15, 32, 33]. Alcohol increases risky sexual behaviours in various populations groups and this relationship is thus not surprising [2–4].

### Implications for policy and practice

These finding stress the need to address the current gaps in condom promotion and supply of lubricants among men who have sex with men in Uganda. Insufficient supply of condoms and lubricants, and misinformation associated with HIV among MSM should be addressed with urgency. The current legal environment poses challenges to the delivery of such interventions. However, the MSM are fairly well organized and several of them participate in social networks that provide an opportunity for mobilization and delivery of HIV prevention interventions [21]. Peers within such networks could be trained to educate these communities and distribute condoms and lubricants.

The underlying factors such as trust and negotiation for condoms should be addressed in educational programs. Current efforts appear to focus on women but clearly some men also need to be empowered to negotiate for safer sex, especially the receptive men in the sex industry.

### Limitations

This study provides some insights into the barriers to condom use among MSM in Uganda. Because this is a highly stigmatized and hidden population, the study participants’ probably belong towards the more open MSM (excluding the closeted groups). This is a potential limitation of our study since results are only transferable to populations in similar contexts. Since we aimed to explore experiences of non-condom users, we did not include experiences of those who did use condoms. This is also a limitation since including men who use condoms could have provided broader view on the phenomenon of condom use in men who have sex with men. Having information on experiences of those men on access, knowledge, partner relationships, alcohol use and finances could provide additional depth to our knowledge to inform interventions. The data for this study was collected in multiple languages, which required translation into a single language for analysis hence, translation bias could have occurred. To minimise language and translation issues, we used interviewers who were fluent in English and local languages and had more than one member of the study team review the transcripts. Despite these limitations, the findings are of practical importance to inform further research and HIV prevention programs among MSM in Uganda.

## Conclusions

There is urgent need to target MSM with interventions designed to promote safer sexual behaviours especially consistent condom use and use of appropriate lubricants, to prevent the acquisition and transmission of HIV and other STIs. Given that MSM are closeted populations and their practices are criminalized, outreach services, drop in centres, and trained peers within the MSM networks could provide opportunities for increasing access to information, condoms and lubricants.

## Supporting Information

S1 Table List of quotations regarding factors influencing non condom use among high risk MSM in Uganda. (DOCX)

## References

[1] Baral S, Sifakis F, Cleghorn F, Beyrer C. Elevated Risk for HIV Infection among Men Who Have Sex with Men in Low- and Middle-Income Countries 2000–2006: A Systematic Review. PLoS Med. 10.1371/journal.pmed.0040339 2007;4(12):e339 18052602 PMC2100144

[2] Koblin BA, Chesney MA, Husnik MJ, Bozeman S, Celum CL, Buchbinder S, et al High-risk behaviors among men who have sex with men in 6 US cities: baseline data from the EXPLORE Study. Am J Public Health. 2003;93(6):926–32. 12773357 10.2105/ajph.93.6.926 PMC1447872

[3] Dudley MG, Rostosky SS, Korfhage BA, Zimmerman RS. Correlates of high-risk sexual behavior among young men who have sex with men. AIDS Educ Prev. 2004;16(4):328–40. 15342335 10.1521/aeap.16.4.328.40397

[4] Lightfoot M, Song J, Rotheram-Borus MJ, Newman P. The influence of partner type and risk status on the sexual behavior of young men who have sex with men living with HIV/AIDS. J Acquir Immune Defic Syndr. 2005;38(1):61–8. 15608527 10.1097/00126334-200501010-00012

[5] Blake SM, Ledsky R, Lehman T, Goodenow C, Sawyer R, Hack T. Preventing sexual risk behaviors among gay, lesbian, and bisexual adolescents: the benefits of gay-sensitive HIV instruction in schools. Am J Public Health. 2001;91(6):940–6. 11392938 10.2105/ajph.91.6.940 PMC1446472

[6] Jin F, Jansson J, Law M, Prestage G, Zablotska I, Imrie J, et al Per-contact probability of HIV transmission in homosexual men in Sydney in the era of HAART. AIDS. 2010;24:907–13. 10.1097/QAD.0b013e3283372d90 20139750 PMC2852627

[7] Patel P, Borkowf CB, Brooks JT, Lasry A, Lansky A, Mermin J. Estimating per-act HIV transmission risk: a systematic review. Aids. 2014;28(10):1509–19. 10.097/QAD.0000000000000298 24809629 PMC6195215

[8] Okal J, Luchters S, Geibel S, Chersich MF, Lango D, Temmerman M. Social context, sexual risk perceptions and stigma: HIV vulnerability among male sex workers in Mombasa, Kenya. Cult Health Sex. 2009;11(8):811–26. 10.1080/13691050902906488 19484638

[9] Baggaley RF, White RG, Boily MC. HIV transmission risk through anal intercourse: systematic review, meta-analysis and implications for HIV prevention. Int J Epidemiol. 2010;39(4):1048–63. 10.93/ije/dyq057 Epub 2010 Apr 20. 20406794 PMC2929353

[10] WHO. Global report: UNAIDS report on the global AIDS epidemic 2013 Geneva: World Health Organisation 2013.

[11] Hladik W, Musinguzi J, Kirungi W, Opio A, Stover J, Kaharuza F, et al The estimated burden of HIV/AIDS in Uganda, 2005–2010. Aids. 2008;22(4):503–10. 10.1097/QAD.0b013e3282f470be 18301063

[12] Hladik W, Barker J, Ssenkusu JM, Opio A, Tappero JW, Hakim A, et al HIV infection among men who have sex with men in Kampala, Uganda—a respondent driven sampling survey. PLoS One. 2012;7(5):e38143 10.1371/journal.pone.0038143 Epub 2012 May 31. 22693590 PMC3364961

[13] Ostergren JE, Rosser BR, Horvath KJ. Reasons for non-use of condoms among men who have sex with men: a comparison of receptive and insertive role in sex and online and offline meeting venue. Cult Health Sex. 2011;13(2):123–40. 10.1080/13691058.2010.520168 20967649 PMC3010288

[14] Li H, Lau JT, Holroyd E, Yi H. Sociocultural facilitators and barriers to condom use during anal sex among men who have sex with men in Guangzhou, China: an ethnographic study. AIDS Care. 2010;22(12):1481–6. 10.080/09540121.2010.482121 20824556

[15] Carballo-Dieguez A, Dolezal C. HIV risk behaviors and obstacles to condom use among Puerto Rican men in New York City who have sex with men. Am J Public Health. 1996;86(11):1619–22. 8916531 10.2105/ajph.86.11.1619 PMC1380700

[16] Smith AD, Tapsoba P, Peshu N, Sanders EJ, Jaffe HW. Men who have sex with men and HIV/AIDS in sub-Saharan Africa. Lancet. 2009;374(9687):416–22. 10.1016/S0140-6736(09)61118-1 Epub 2009 Jul 17. 19616840

[17] King R, Barker J, Nakayiwa S, Katuntu D, Lubwama G, Bagenda D, et al Men at Risk; a Qualitative Study on HIV Risk, Gender Identity and Violence among Men Who Have Sex with Men Who Report High Risk Behavior in Kampala, Uganda. PLoS One. 10.1371/journal.pone.0082937 2013;8(12):e82937 24358239 PMC3866199

[18] Raymond HF, Kajubi P, Kamya M, Rutherford G, Mandel J, McFarland W. Correlates of Unprotected Receptive Anal Intercourse Among Gay and Bisexual Men: Kampala, Uganda. AIDS and Behavior. 2009 2009/08/01;13(4):677–81. 19495955 10.1007/s10461-009-9557-7 PMC2829255

[19] Hladik W, Barker J, Ssenkusu JM, Opio A, Tappero JW, Hakim A, et al HIV Infection among Men Who Have Sex with Men in Kampala, Uganda–A Respondent Driven Sampling Survey. PLoS One. 10.1371/journal.pone.0038143 2012;7(5):e38143 22693590 PMC3364961

[20] Mishra SR, Khanal V. Sexual behaviors among men who have sex with men: a quantitative cross sectional study in Kathmandu Valley, Nepal. Hiv Aids. 2013;5:81–8. 10.2147/HIV.S42795 PMC3639115 23637561

[21] Geofrey Musinguzi HB, Nuwaha Fred, Mujisha Geoffrey, Kiguli Juliet, Arinaitwe Jim, Van geertruyden JP, Wanyenze Rhoda. Barriers to and opportunities for accessing HIV services among Ugandan MSM: a qualitative study International AIDS Society 2014.

[22] Gysels M, Pool R, Bwanika K. Truck drivers, middlemen and commercial sex workers: AIDS and the mediation of sex in south west Uganda. AIDS Care. 2001;13(3):373–85. 11397339 10.1080/09540120120044026

[23] SAGE Publications I. Encyclopedia of Social Science Research Methods. Encyclopedia of Social Science Research Methods. SAGE Publications, Inc. Thousand Oaks, CA: SAGE Publications, Inc.

[24] Morse JM. Confusing categories and themes. Qual Health Res. 2008;18(6):727–8. 10.1177/1049732308314930 18503013

[25] Vaismoradi M, Turunen H, Bondas T. Content analysis and thematic analysis: Implications for conducting a qualitative descriptive study. Nursing & Health Sciences. 2013;15(3):398–405. 23480423 10.1111/nhs.12048

[26] Fields EL, Bogart LM, Smith KC, Malebranche DJ, Ellen J, Schuster MA. HIV risk and perceptions of masculinity among young black men who have sex with men. J Adolesc Health. 2012;50(3):296–303. 10.1016/j.jadohealth.2011.07.007 Epub Oct 2. 22325136 PMC3281559

[27] Pando MA, Balan IC, Marone R, Dolezal C, Leu CS, Squiquera L, et al HIV and other sexually transmitted infections among men who have sex with men recruited by RDS in Buenos Aires, Argentina: high HIV and HPV infection. PLoS One. 2012;7(6):e39834 10.1371/journal.pone.0039834 Epub 2012 Jun 29. 22768137 PMC3387227

[28] Adams J, Neville S. Men Who Have Sex With Men Account for Nonuse of Condoms. Qualitative Health Research. 2009 December 1, 2009;19(12):1669–77. 10.1177/1049732309353046 19949217

[29] Tucker S, Krishna R, Prabhakar P, Panyam S, Anand P. Exploring dynamics of anal sex among female sex workers in Andhra Pradesh. Indian J Sex Transm Dis. 2012;33(1):9–15. 0.4103/0253-7184.93787. 10.4103/0253-7184.93787 22529447 PMC3326863

[30] Alexander M, Mainkar M, Deshpande S, Chidrawar S, Sane S, Mehendale S. Heterosexual Anal Sex among Female Sex Workers in High HIV Prevalence States of India: Need for Comprehensive Intervention. PLoS One. 10.1371/journal.pone.0088858 2014;9(2):e88858 24586416 PMC3930672

[31] Varga CA. The condom conundrum: barriers to condom use among commercial sex workers in Durban, South Africa. Afr J Reprod Health. 1997;1(1):74–88. 10214405

[32] Vittinghoff E, Douglas J, Judson F, McKirnan D, MacQueen K, Buchbinder SP. Per-contact risk of human immunodeficiency virus transmission between male sexual partners. Am J Epidemiol. 1999;150(3):306–11. 10430236 10.1093/oxfordjournals.aje.a010003

[33] Musinguzi G, Bwayo D, Kiwanuka N, Coutinho S, Mukose A, Kabanda J, et al Sexual Behavior among Persons Living with HIV in Uganda: Implications for Policy and Practice. PLoS One. 10.1371/journal.pone.0085646 2014;9(1):e85646 24465631 PMC3900429

