# Supplementary material for: Barriers to Condom Use among High Risk Men Who Have Sex with Men in Uganda: A Qualitative Study
Source: PLoS One. 2015 Jul 14;10(7):e0132297. doi: 10.1371/journal.pone.0132297 (PMC4501754; doi:10.1371/journal.pone.0132297)
Supplement: S1 Table — (DOCX) [file pone.0132297.s001.docx]

**Annex**

**List of quotations regarding factors influencing non condom use among high risk MSM in Uganda**

______________________________________________________________________

- **Condom related factors and access factors**

______________________________________________________________________

- ***Condom lodging and the perceived homophobic healthcare environment***

***List of quotations***

- …fear that this condom will go and enter into your “whatever” and can’t come out! And where will you go?” “…it continues and when you are taken for treatment. What will you say? They will ask you, “How has it entered? What have you been doing?” (IDI 1 Bushenyi).
- …I just don’t like using condoms because when I had of cases where a condom could stick in the anus I am imaging if it stuck there where will I run to and how will people hear that a condom stuck in my anus, so such things make me not like condoms and imagine going to the doctor who is not used to such things (IDI 7 Kampala).
- The fear of a condom to stick in the anus that one hinders. (IDI 3 Bushenyi)
- **Poor Quality condoms**

***List of quotations***

- Sometimes you use condoms like hard condoms. So they break during sex. Sometimes it depends on the Lubricant you are using... So you end up using like three condoms and they are all bursting (x3). Let me say I have un protected sex, however much I try like doing protected sex and the condom breaks (IDI 7 Kampala)
- Sometimes they break …Some are not of good quality, that is; anal sex is a bit rough so we need to be having a good quality condom (he pauses) because these things are a bit rough. (IDI 7 Kampala)
- um, I actually can’t give you a reason as to why am not using a condom, because, at first I tried using a condom, it actually burst (IDI Mbarara 3)
- we had engabo (condom type) they were telling us that they are not safe , you know all those issues, we heard complaints about condoms, so I believe not using a condom, may be is better (IDI 3 Mbarara)
- I think that’s due to being expired, some are not of good quality , that is; anal sex is a bit rough so we need to be having a good quality condom (he pauses)because these things are a bit rough. (IDI 7 Kampala)

?? **Lubricants sufficient**

***List of quotations***

- We didn’t use a condom but we had avis (IDI 4 Iganga)
- Why don’t you use a condom when having sex with men? I usually use lubricants and saliva (IDI 7 Iganga)
- While having sex with fellow men we use lubricants to ease the penetration; hence I think the condom is not necessary (IDI 7, Iganga)
- We are given lubricants and if we have tested and we both know our status there is no need for the condom. (IDI 10, Mukono)
- **Irritation and pains**

***List of quotations***

- Any way we also just use condoms to protect lives but it is so painful. (IDI 5, Bushenyi).
- The use of condom is not bad but the problem is when used for more than a minute, it tends to get dry and it starts hurting and even it can create bruises. It can only be good when one uses for a few minutes, then get a new one. So it makes the whole process tedious and not enjoyable (IDI 3 Soroti)
- Some men don’t want condoms claiming that they are irritating. Any way we also just use condoms to protect lives but it is so painful. (IDI 2 Kampala)
- **Access factors**
- **Physical access challenges**

***List of quotations***

- And sometimes they run out of them. (IDI 7 Kampala)
- We had nowhere to get them from. (IDI 3 Iganga)
- We sometimes don’t use those things because we don’t have them (IDI 6 Iganga)
- Because these things you see here [condom] I even never had them, I got this one from …; because if I had them I wouldn’t experience that damage (IDI 6 Iganga)
- and you know, like some guys deep in the villages, they would really want to buy condoms but where to buy them,
- The condoms are there but ours are not there, we as the gays (IDI 4 Busia)
- lack of commodities like condoms (IDI 2 Gulu)
- failing some times to get lubricants and the condoms (IDI 7 Hoima)
- Now you just imagine who can stand waiting till those condoms and lubes are available? (IDI 4 Mukono).
- condoms are not there in supply (IDI 1 Soroti)
- **No money to buy condoms**

***List of quotations***

- At times when the condoms are not available and you don’t have money to buy them (IDI 1 Kampala)
- Someone could want to protect themselves but because of lack of those condoms and you find someone doing it without a condom (IDI 7 Mbarara)
- The challenges are that at times you may want to have sex yet you do not have money to buy condoms. (IDI 5 Kampala)
- Sometimes you might not be having condoms and yet you even have no money to buy them (IDI 2 Bushenyi)

______________________________________________________________________

- **Knowledge and perception related factors**

______________________________________________________________________

***List of quotations***

- I don’t quite understand how they work (IDI 8 Mbarara)
- it is because I have never used it before. (IDI 3 Rakai)
- I did not know anything about them. (Iganga IDI 3)
- Some people fear them. (Hoima IDI 8)
- I don’t quite understand how they work and sometimes when you get together with someone and you have been together for a while you find no need of using a condom (IDI 8 Mbarara)
- also the other obstacle around a round is the lack of knowledge about the use of condoms among the MSM (IDI 1 Mbale)
- **Altered messages by mass media and some members in the MSM network**

***List of quotations***

- The sensitizations we hear over the radios say condoms cause diseases (IDI 2 Iganga)
- They say that even if you protect yourself that much instead you get HIV infection and even diseases like Candida plus many other diseases are due to condom lubrications (IDI 2 Iganga)
- They also bring condoms and give them to us but they warn us in advance that we shouldn’t lie ourselves that we have sex with women while putting on condoms in order not to acquire HIV instead we will acquire it, that’s one of the reasons we say we can’t prevent it. (IDI 2 Iganga).
- Diseases like Candida plus many other diseases are due to condom lubrications (IDI 2 Iganga)
- The condoms can’t prevent HIV (IDI 2 Iganga)
- condoms can’t prevent HIV but they can prevent pregnancy in case you have sex with a woman but they can’t prevent AIDS (IDI 2 Iganga)
- **Perception that HIV is not transmitted in a sexual relationship between men**

***List of quotations***

- I did not use a condom reason being that wasn’t having sex with woman
- But one way of eradicating HIV is avoiding having sex with women but only to have it with fellow men. …but here in a sexual intercourse of man with a fellow man, a man releases alone and you see this is factor that prevents HIV here because it is only one person releasing the sperms you have no chances of getting infected with HIV. (IDI 2 Iganga)
- I have a feeling that a homosexual never contracts HIV
- There are those also who think that someone having sexual intercourse with a fellow man cannot get HIV with the reasoning that the man does not have the fluid like the ladies and yet they have always known that it is transmitted through fluids so how do they get it. (IDI 1 Mbale)
- **Oral sex not suitable with a condom**

***List of quotations***

- There’s also this other business of sucking one’s penis but again I don’t think it is healthy to suck when someone has put on a condom you will be sucking condom components which I don’t think are healthy” (IDI 3, Bushenyi)

___________________________________________________________________

- **Partner and relationships related factors**

___________________________________________________________________

- ***Trust***

***List of quotations***

- I used it in the beginning and after knowing and getting used to my partner I stopped using it and it is because I felt like I didn’t want to use it any more. (IDI 7 Hoima)
- We are not close as partners but he told me that he was safe so I decided to trust him and he also trusted me. (IDI 6 Mbale)
- you’re like I have been with someone for long (IDI 2 Mbale)
- I think the best way of protection is trust. (IDI 8 Kampala)
- you have known each other for some time (IDI 8 Busia)
- No, I think it’s only because we trusted each other (IDI 6 Mbale)
- It’s because we have been together for a long time. (IDI 5 Mbarara)
- We had trusted each other I have spent a long time with this partner, why do I need to use protection? (IDI 8 Mbarara)
- I didn’t use a condom in the last sexual intercourse I had because I trusted the person I was with (IDI 2 Mukono)
- I have been with this partner for some time we trust each other, sometimes we don’t normally use condoms we just trust each other (IDI 7 Kampala)
- Sometimes when you get together with someone and you have been together for a while you find no need of using a condom. (IDI 8 Mbarara)
- you may get someone who doesn’t want to use those things when you are also in moods and you don’t have to force it. (IDI 7 Busia)
- **Trust embedded with knowledge of HIV status**

***List of quotations***

- I never used a condom because I trust my partner, we always together and we checked and tested for HIV together and got to know that we are safe from HIV virus. So we are not worried about each other. (IDI 3 Soroti)
- No because I have been with him for two and a half years, at first we were using condoms but then we had to go for an HIV test so after that test we tested negative so we trust each other. Like after three months, we go for a test. (IDI 7 Mbarara)
- **Is it trust or weaker safer sexual negation skills?**

***List of quotations***

- I remember there is a time I tried to speak about using it then he was like objecting it; he asked me that “you no longer trust me now days?” and because I love him I have to accept to go without any protection (IDI 4 Mbale)
- We are not close as partners but he told me that he was safe so I decided to trust him and he also trusted me. (IDI 6 Mbale)
- The person I used to have sex with didn’t use a condom (IDI 1 Mukono)
- He doesn’t want to use it (IDI 2 Rakai)
- For me I would like to use it but for him he doesn’t want to use it, I don’t know why? (IDI 2 Rakai)
- He did not want to use one. (IDI 3 Rakai)
- Sometimes I didn’t use a condom some men would have sex with me without a condom
- Sometimes you endure (IDI 8 Busia)
- **None cooperative and sometimes violent partners**

***List of quotations***

- Sometimes you may find that you have got a client who does not want to use a condom and he convinces you to have sex with him without using a condom (IDI 1 Busia)
- You can get a client who doesn’t want to use a condom so much as you negotiate over condom use he can pretend as if he has put it on and instead tears it in interest of enjoying live sex (IDI 6 Busia)
- There those Kuchus who have a lot of appetite so when you tell them to protect themselves they don’t want (IDI 3 Hoima)
- On other occasions you as for one may prefer putting on a condom so that you have a safe sexual intercourse but your partner may not want to use them. (IDI 5 Kampala)
- partners simply do not want condoms because they have a reasoning that a couple that uses a condom have no faith in each other (IDI 1 Mbale)
- then some other men want to force you (IDI 4 Mbarara)
- **Unplanned sexual intercourse**

***List of quotations***

- It was not ready, it was not available (IDI 1 Bushenyi)
- I had not prepared it. (IDI 1 Bushenyi)
- Sometimes it happens let’s say in the night when you do not have condoms in the house so you end up doing it (IDI 7 Kampala)
- I always use a condom, that’s the only mistake I made. (IDI 1 Mbarara)
- A fellow MSM can put you in moods yet you don’t have condoms so you find yourselves having sex without them [condoms] (IDI 2 Bushenyi)
- Sometimes you have stayed without getting a client and at the time you get one, you might not easily get the condoms and if it finds that the client is in a hurry you will end up having sex without a condom. (IDI 1 Busia)
- some maybe get strong urges and find that they have no time to go buy a condom (IDI 8 Hoima)
- someone just calls you, you are expecting just a friendly chat and you find you are having unprotected sex with that person. (IDI 7 Mbarara)
- **Attractiveness of the partner**

***List of quotations***

- you may admire him because he is handsome and you decide to do it (sex) live thinking that he may be negative. (IDI 1 Hoima)
- There are times when you find a very attractive woman and you decide not to wear a condom, (IDI 1 Hoima)
- seeing someone and you’re like this one is hot and this self control isn’t meant for everyone (IDI 1 Mbarara)

______________________________________________________________________

- **Money power and socio economic vulnerability**

***List of quotations***

- The man was giving me a lot of money and he said he never wanted a condom
- Yes he said that if he gets live sex he will give me more money and if it’s with a condom less money and I accepted to have live sex (IDI 3 Kampala)
- You may find that he tells you am paying at this term with a condom and without a condom am paying such a term, and just because I need money. The first sum without a condom and with a condom, thirty ,and you may find that you are having that client today but it will take you something like a week so you think about that and say you need the money so you will be forced to go for the bigger amount. And remember I have very many needs I have to pay my rent dress up and eat. (IDI 5 Busia)
- The charges also change when someone does it (sex) live and when someone wears a condom. If it is live, the fee is higher. (IDI 1 Kampala)
- one who uses can give you 10000ugx but if he doesn’t, he can give you more (IDI 1 Kampala)
- but if the customer does not want to use a condom i charge him higher – between 250,000/= and 300,000/= and with a condom on it is between 150,000/= and 180,000/=. (IDI 2 Gulu)

______________________________________________________________________

- **Alcohol and other factors (Exploring, excitement, maximizing pleasure and hurry)**

_____________________________________________________________________

- **Alcohol**

***List of quotations***

- There are times when you take a lot of alcohol, and when you take a lot of alcohol you will become sexually very active and may not even think of using a condom (IDI 2 Busia)
- When you are drunk and you reach home and you are so … you may find yourself doing it without a condom (IDI 5 Busia)
- Now when you’re drunk because when you are drunk you end up doing something and tomorrow you’re like I forgot to use a condom (IDI 2 Mbale)
- The only challenge is like people taking alcohol and out of this; they start having unprotected sex. (IDI 1 Mbarara)
- When you are alcoholic and taking drugs. (IDI 10 Mukono)
- You may get drunk and you end up having unprotected sexual intercourse that would not have done when sober and you regret. (IDI 10 Mukono)
- Some MSMs use drugs and alcohol which affect their normal reasoning capacity (IDI 4 Hoima)
- **Pleasure maximization, exploration and being in a hurry**

***List of quotations***

- You know as you are eating a sweet from its cover, it cannot be the way it should be enjoyed, therefore I just move! (IDI 1 Bushenyi)
- Another thing is that when I use a condom, I’m not comfortable when having sex. And I don’t feel the person well. And actually many people don’t want to use condoms because they don’t feel well with it while having sex. And with a condom on, I don’t enjoy sex
- Maybe it was sexual curiosity and sexual interest (IDI 1 Bushenyi)
- It makes you play quick sex without that condom. (IDI 1 Bushenyi)
- Maybe like I told you earlier, it is sexual interest; because you want to move quickly or not be seen by somebody, so you say, let me do it in the fastest way instead of waiting for a condom. (IDI 1 Bushenyi)
- Sometimes when you’re over excited you end up not protecting. (IDI 2, Mbale)
- impatience; seeing someone and you’re like this one is hot and this self control isn’t meant for everyone (IDI 1 Mbarara)
- strong and uncontrollable desire for the partner (IDI 7 Rakai)
- You can get a man when you’re also in moods and he tells you that he doesn’t want to use a condom (IDI 7 Busia)

**Exchanging condoms for sex**

Quotation

But, at times, the leaders (of the organization) say if you really want to get condoms, lubricants, you have to go in with me. And at times you don’t feel like you like this very person and you really find difficulties going in with him, but just because you really want what you want, you go in with them. (IDI 3 Mbarara)

______________________________________________________________________
